# Editorial: Health-stress-disease triangle. Pathophysiological focus and perspectives

**DOI:** 10.3389/fphys.2024.1427085

**Published:** 2024-05-17

**Authors:** Claudia Capurro, Luis Sobrevia, Maria Cecilia Larocca, Graciela Alicia Cremaschi, Martin Vila Petroff

**Affiliations:** ^1^ Departamento de Ciencias Fisiológicas, Laboratorio de Biomembranas, Facultad de Medicina, Instituto de Fisiología y Biofísica “Bernardo Houssay” (IFIBIO-HOUSSAY), Consejo Nacional de Investigaciones Científicas y Técnicas (CONICET), Universidad de Buenos Aires, Buenos Aires, Argentina; ^2^ Cellular and Molecular Physiology Laboratory (CMPL), Division of Obstetrics and Gynaecology, School of Medicine, Faculty of Medicine, Pontificia Universidad Católica de Chile, Santiago, Chile; ^3^ Tecnologico de Monterrey, Institute for Obesity Research, School of Medicine and Health Sciences, Monterrey, Mexico; ^4^ Department of Physiology, Faculty of Pharmacy, Universidad de Sevilla, Seville, Spain; ^5^ Medical School (Faculty of Medicine), São Paulo State University (UNESP), São Paulo, Brazil; ^6^ Faculty of Medicine and Biomedical Sciences, University of Queensland Centre for Clinical Research (UQCCR), University of Queensland, Herston, QLD, Australia; ^7^ Instituto de Fisiología Experimental (IFISE) - Consejo Nacional de Investigaciones Científicas y Técnicas (CONICET), Facultad de Ciencias Bioquímicas y Farmacéuticas–Universidad Nacional de Rosario, Rosario, Argentina; ^8^ Laboratorio de Neuroinmunomodulación y Oncología Molecular, Instituto de Investigaciones Biomédicas (BIOMED), Pontificia Universidad Católica Argentina (UCA), Consejo Nacional de Investigaciones Científicas y Técnicas (CONICET), Ciudad Autónoma de Buenos Aires, Argentina; ^9^ Centro de Investigaciones Cardiovasculares Horacio Cingolani, CONICET La Plata, Facultad de Ciencias Médicas, Universidad Nacional de La Plata, La Plata, Argentina

**Keywords:** cardiovascular physiology, inflammation, traslational medicine, heart, hypertension

This Frontiers Research Topic emerged as an outcome of the 2022 annual joint meeting of the Argentinean Society of Physiology (SAFIS) and the Latin American Association of Physiological Sciences (ALACF). Targeted to highlight physiological and pathophysiological studies focused on the interrelation between health, stress and disease and the molecular pathways that either favour, break or reverse this unidirectional path. The articles published herein reveal groundbreaking research on the response of the cardiovascular system to diverse stress signals.

Basic and clinical studies have proved the efficacy of anti-inflammatory therapies in the prevention of cardiovascular (CV) diseases of different aetiology. Accordingly, inflammation has recently emerged as a potential mediator of CV disease. However, despite numerous investigations highlighting the causal role of inflammation in cardiovascular pathophysiology, inflammation is an extremely complex phenomenon in which specific cellular mechanisms of action remain elusive. In this Research Topic, the review article by Seropian et al. focuses on the critical cardiovascular effects of the inflammatory mediator Galectin-3 (Gal-3) and sheds light on its potential therapeutic benefit to reduce CV disease. Gal-3 belongs to the family, of beta-galactoside-binding proteins, and is involved in many biological processes including inflammation. The role of Gal-3 in inflammation is multifaceted and paradoxical as both pro-inflammatory and anti-inflammatory responses have been reported. However, Gonzalez et al. compellingly clarify the diversity of inflammatory actions of Gal-3 in the pathological settings of myocardial infarction, hypertension, atherosclerosis, and aging. Although the available literature provides some contradictory results, the consensus points to Gal-3 as a pro-inflammatory molecule with an active role in the development of fibrosis, atherosclerotic plaque, adverse remodelling, and myocardial contractile dysfunction. In this scenario, Gal-3 inhibitors emerge as innovative cardiovascular therapeutic agents with clinical perspectives.

Pro- and anti-inflammatory responses depend not only on the molecular nature of the inflammatory mediator but also on the balance between pro- and anti-inflammatory immune cells. Thus, cell-to-cell interaction arises as a pivotal player shaping the inflammatory response. An exquisite example of this intricate interplay is revealed in the article published by Lantz et al. and colleagues where the altered balance between pro-inflammatory T helper 17 (TH17) cells and anti-inflammatory regulatory T cells (Tregs) is shown to orchestrate a pro-inflammatory microenvironment that is essential for the development of pulmonary hypertension (PH). The study demonstrates that in response to chronic hypoxia there is a switch of Treg to TH17 cells, denominated exTreg-TH17 cells, which tilts the Treg–TH17 cell balance toward TH17 cells, creating a pro-inflammatory environment. These results suggest that therapies aimed at restoring active Tregs and preventing the transition of exTreg-*to*-TH17 cells could be a potential avenue for the treatment of PH.

Ischemic heart disease constitutes a major cause of death worldwide and in this pathological setting, inflammation has also been shown to play a critical role. Similarly, reactive oxygen species (ROS) contribute significantly to ischemia-reperfusion (IR) injury. Ischemic preconditioning is a strategy whereby brief episodes of ischemia by occlusion of a coronary artery followed by reperfusion, activate endogenous protective mechanisms and decrease IR damage. Cardiac protection also occurs when brief preconditioning ischemic episodes occur in a distant tissue, such as a limb, which is termed remote ischemic preconditioning (RIPC). Multiple lines of evidence indicate that inhibition of NLR family pyrin domain containing 3 (NLRP3)-inflammasome-dependent inflammation and NADPH oxidase type 2 (NOX2)-dependent ROS production prevents ischemic preconditioning. However, there is scarce information on the participation of these pathways in RIPC. In this Research Topic issue of Frontiers in Physiology, the study by Benavides et al. convincingly demonstrates that RIPC activates NOX2 and the NLRP3 inflammasome resulting in a secondary antioxidant and anti-inflammatory response that confers protection against myocardial IR injury. In depth understanding of the molecular mechanisms of cardiac protection as those shown in this Research Topic issue, pave the way to the achievement of novel protective protocols that may prove beneficial for the treatment of patients exposed to IR injury.

Another critical player in IR injury is intracellular Ca^2+^ overload. Enhanced Ca^2+^ entry via the L-type Ca^2+^ channels is a major contributor to this cation overload. The study of the impact of Ca^2+^ channel inhibitors on IR injury is a vibrant and controversial area of research. Inhibition of Ca^2+^ channels has been shown to be effective to ameliorate post-ischemic damage in animal and cellular models of IR. However, the clinical use of Ca^2+^ channel blockers in myocardial infarct is still in dispute because of their marked hemodynamic effects. Thus, the search for novel and safer compounds that specifically interfere with Ca^2+^ entry is ongoing. In this Research Topic, Pardo et al. discuss the use of an alternative compound, N-methylacetazolamide (NMA), with potential benefits to those or classical Ca^2+^ channel antagonists. NMA is derived from the carbonic anhydrase (CA) inhibitor acetazolamide. The substitution of a methyl group for an H^+^ in the sulfonamide moiety of acetazolamide results in a loss of its CA inhibitory property while maintaining the physical-chemical properties of acetazolamide. In animal models of cardiac IR, NMA was shown to significantly reduce infarct size and diastolic stiffness and to increase systolic function. These beneficial effects were attributed to a direct inhibitory effect of NMA on the pore of the Ca^2+^ channel. Although the mechanism of action of NMA seems to be shared with classical L-type Ca^2+^ channel blockers, the fact that NMA was shown to have protective features even when given during reperfusion represents an attractive alternative to ameliorate post ischemic impairment. This possibility makes NMA a unique tool for the treatment of ischemic heart disease in patients. Future clinical trials will be mandatory to demonstrate the effectiveness of NMA in humans and its advantages or disadvantages in comparison to other Ca^2+^ antagonists. Considering the causal role of inflammation in IR injury, and the existing evidence showing that Ca^2+^ overload triggers inflammation, further investigation examining the capacity of NMD to mitigate inflammation is warranted.

As we dive into the depth of the physiological findings of the articles published in this Research Topic, the important contribution of SAFIS and ALACF to the knowledge of this disciplinary area is reflected. As we transition from one article to the next, we immerse ourselves in groundbreaking research, fostered by international collaboration and enriched by both basic and clinical perspectives. Each page breaths an educational atmosphere, inviting readers to explore new physiological discoveries and push the boundaries of our understanding.

